# The social lives of isolates (and small language families): the case of the Northwest Amazon

**DOI:** 10.1098/rsfs.2022.0054

**Published:** 2022-12-09

**Authors:** Rik Van Gijn, Sietze Norder, Leonardo Arias, Nicholas Q. Emlen, Matheus C. B. C. Azevedo, Allison Caine, Saskia Dunn, Austin Howard, Nora Julmi, Olga Krasnoukhova, Mark Stoneking, Jurriaan Wiegertjes

**Affiliations:** ^1^ Leiden University Centre for Linguistics, Leiden 2311 BE, The Netherlands; ^2^ Copernicus Institute of Sustainable Development, Environmental Science Group, Utrecht University, Utrecht 3584 CB, The Netherlands; ^3^ Department of Evolutionary Genetics, Max-Planck-Institute for Evolutionary Anthropology, Leipzig 04103, Germany; ^4^ University of Groningen, Campus Fryslân 8911 CE, The Netherlands; ^5^ Department of Anthropology, University of Wyoming, 82071, Laramie, WY, USA; ^6^ Université Lyon 1, CNRS, Laboratoire de Biométrie et Biologie Evolutive, UMR 5558, Villeurbanne, France

**Keywords:** social history, language isolates, Northwest Amazon

## Abstract

The Americas are home to patches of extraordinary linguistic (genealogical) diversity. These high-diversity areas are particularly unexpected given the recent population of the Americas. In this paper, we zoom in on one such area, the Northwest Amazon, and address the question of how the diversity in this area has persisted to the present. We contrast two hypotheses that claim opposite mechanisms for the maintenance of diversity: the isolation hypothesis suggests that isolation facilitates the preservation of diversity, while the integration hypothesis proposes that conscious identity preservation in combination with contact drives diversity maintenance. We test predictions for both hypotheses across four disciplines: biogeography, cultural anthropology, population genetics and linguistics. Our results show signs of both isolation and integration, but they mainly suggest considerable diversity in how groups of speakers have interacted with their surroundings.

## 1. Introduction

**Figure 1.**
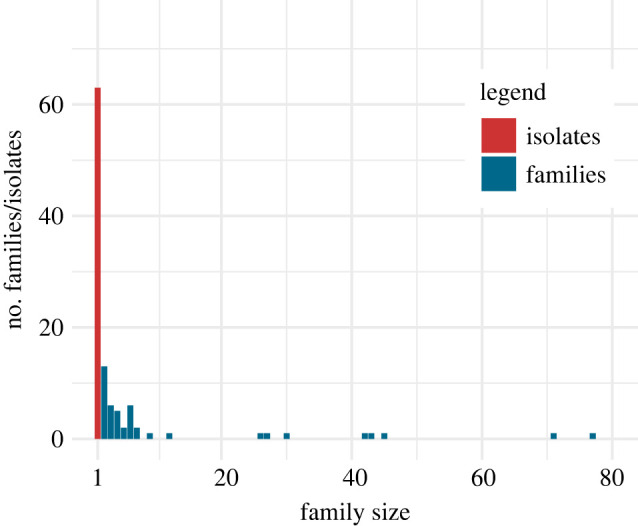
Size of language families in South America. For families of a given size (*x*-axis), it is shown how many families fall within that group (*y*-axis).

The linguistic landscape of South America presents an intriguing paradox. On the one hand, there is broad consensus that humans populated the Americas via the temporary land bridge between Siberia and Alaska relatively recently: some 15 000–20 000 years ago. (Although the precise timeline and routes remain unclear, this final leg in the human population of the world is largely supported in general terms by geological [1,2], genetic [3,4] and archaeological [5,6] evidence.) On the other hand, this shallow time depth is difficult to reconcile with the continent's profound linguistic diversity, in particular genealogical diversity (i.e. the number of language families). While taking up about 13% of the Earth's inhabitable land mass, South America is home to slightly fewer languages than expected compared to global distributions^1^ (about 8% of the world's languages), but, unexpectedly, these languages belong to more genealogical lineages than most other areas in the world (containing representation of 27% of recognized maximally reconstructible language families).^2^ Most of these language families are very small, consisting of two or three known surviving members, or even just one (known as isolates). In fact, South America contains 64 isolates, 34% of the global tally. Figure 1 gives the frequency distributions of families of different sizes in South America, showing the abundance of isolates.

The extraordinary genealogical diversity of South America's linguistic landscape, and in fact of the Americas as a whole,^3^ is not straightforwardly reconcilable with the continent's relatively recent initial population by humans. Focusing on the Americas as a whole, Nichols [8] calculated, based on models of rates of change from well-studied language families elsewhere in the world, that it would take about 35 000 years for the level of genealogical diversity found in all of the Americas to develop. This apparent contradiction between high linguistic diversity and relatively recent population, in combination with a population bottleneck, has become known in the literature as the American puzzle, or the American paradox [8–11].

Starting as early as 1916, with Sapir's discussion of the linguistic diversity of the Americas [12], two main proposals have been offered to resolve this paradox. The first suggests that the population that first populated the Americas was already linguistically diverse at the time of entry [8].^4^ A second proposal suggests that in the initial stages of the peopling of the Americas, the linguistic diversification rates were higher, and the number of fissioning events were more numerous, leading to a more rapid development of separate linguistic lineages [9].^5^ It is also possible that both of these factors contributed to the genealogical diversity of the continent's present linguistic panorama.

A related question, which has received less attention in the literature, is how this accumulated diversity has survived (albeit in patches) until the present day. This is an important part of understanding the American paradox, because explaining the continent's high degree of linguistic diversity requires attention not only to the processes by which it was generated, but also to the processes by which it has been retained. This question is the focus of the present paper. We approach this question by bringing together two partly competing hypotheses, inspired by proposals from Nettle [9] and Epps [11], which we will term the *isolation hypothesis* and the *integration hypothesis*, respectively.

Nettle proposes a model both for the emergence and the persistence of genealogical diversity in the Americas. A crucial observation in Nettle's proposal is that global linguistic history is not one of ever-increasing, regular diversification. If so, we would expect Africa and Eurasia, places with longer population histories, to be more diverse than Oceania, Australia and the Americas, which were populated more recently. However, this is clearly not the case when it comes to genealogical units. Instead, the long-term trend seems to be a *decrease* in genealogical diversity [9]. To account for this, as well as for the high degree of diversity in the Americas, Nettle proposes, based on a version of the punctuated-equilibrium model of Dixon [20], that diversity increases as the result of major demographic events (punctuations), until the available space for further rapid differentiation becomes limited. After this point, net diversity decreases, because linguistic lineages become extinct as populations come into ever closer and more intense contact.

For Nettle, the initial colonization of the Americas represents a special punctuation event, whose diversification effects went on relatively unimpeded for a long period because of the massive space available. According to Nettle, this allowed ‘groups of foragers [to] spread and fission at a very high rate, as they moved out through the continent (…) It would seem that the Americas in 1492, with their extraordinary stock diversity, were either at the peak or still in the steep rise [of the model]’ [9, pp. 3327–3328].

Of course, rather than phasing out, a punctuated equilibrium can also be interrupted by another punctuation that precipitates a new set of demographic changes. In fact, a second punctuation that leads to a dispersal of populations may speed up lineage extinction as expanding groups incorporate or extinguish existing groups. The emergence of agriculture and the ensuing population growth and dispersals are widely considered to be responsible for supplanting a considerable part of the previously existing linguistic diversity associated with hunter–gatherer groups all over the world [21,22]. This raises the question of why high lineage diversity persists in the Americas, given that the advent of agriculture was roughly contemporaneous with other continents where much less diversity survives. As Jared Diamond writes [23, p. 370]:^6^

Had any food-producing Native American peoples succeeded in spreading far with their crops and livestock and rapidly replacing hunter–gatherers over a large area, they would have left legacies of easily recognized language families, as in Eurasia, and the relationships of Native American languages would not be so controversial.

To be sure, language/agriculture dispersals as described by Diamond are in fact present in the Americas. Language families such as Uto-Aztecan, Arawakan and Tupian have spread over large territories, and these expansions have been (although not always uncontroversially) associated with agricultural activities [22]. But the considerable patches of land with high genealogical diversity are not found in Eurasia. The main point of the quote from Diamond is that the expanding families in the Americas were less extensive than in Eurasia.

Among the reasons^7^ to account for this difference, Diamond [23] and Diamond & Bellwood [22] have suggested that the movement of people and crops (and everything associated with that movement, including plants, animals, technology and innovations) was more difficult in the Americas than in Eurasia because of biogeographic differences. In Eurasia movements followed an east–west axis, while the Americas have a major north–south axis, spanning more ecological environments to which agricultural techniques and crops must adapt. Furthermore, the Americas have a more fragmented ecological panorama, where areas suitable for agriculture are separated by intervening areas which are less suitable for agriculture.

According to Nettle's and Diamond's views, the patches of South America that exhibit a high concentration of isolates and small language families would represent a legacy of that initial punctuation event. In this view, these areas would not have undergone the sort of reduction in genealogical diversity that we find in parts of the world where human presence is older and where language/agriculture dispersals have reduced the degree of linguistic diversity. If this scenario (i.e. the *isolation hypothesis*) is correct, then we would expect to find certain signatures in the linguistic–typological, genetic and sociocultural panoramas of those populations. In particular, these small languages and their speakers should show signs of isolated development until relatively recently (if, at the time of European colonization, the diversification resulting from the initial population was at its peak or still on the rise), and throughout most of their history.

A second influential hypothesis regarding the persistence of South America's unexpectedly diverse linguistic panorama comes from Patience Epps, which we refer to as the *integration hypothesis* [11].^8^ Epps argues against the idea that biogeography had a substantial role in shaping the patterns of diversity found in the Amazon Basin today. For one, there are relatively few natural obstacles in the region (note that rivers, which may in some circumstances function as obstacles, have often been used as conduits of migration and trade in the past). Furthermore, Epps argues against agriculture as a force shaping the patterns of diversity in the Amazon Basin, since most groups practice some form of agriculture. In addition, based on patterns found in other regions of the globe, she argues that agriculture is neither a necessary nor a sufficient condition for language spread. Epps further argues against the idea that high genealogical diversity is necessarily the result of isolation. She points to studies [24–27] indicating that Amazonia ‘was home to areas of dense population and extensive regional formations' [11, p. 273].

Instead, turning the proposals of Nettle and Diamond on their heads, Epps argues that contact and interaction between language groups has driven the preservation rather than the disappearance of linguistic diversity [11]. Her study zooms in on the regional system of the Upper Rio Negro, a linguistically diverse area which is characterized by intense interaction among languages combined with a conscious effort by their speakers to maintain linguistic and cultural differences as markers of social identity. Thus, the groups of the Upper Rio Negro and Vaupés areas have shown considerable resistance to language shift, but also persistence in culturally diverse practices regarding marriage (exogamy versus endogamy) and subsistence strategies (predominantly agriculture-based versus a focus on hunting and gathering). More importantly, the speakers of various languages in the region maintain and amplify particular cultural practices that differentiate them from each other and bind them together in a system of complementarity. These practices include trade and ritual specialization, as well as access to particular ecological niches, both of which are expressed through identity and ethnicity. In this way, groups actively maintain differences not in spite of the regularity of their integration, but because of it. The maintenance of diversity is fundamental to the functioning of such a regional system.

However, this is not to say that all aspects of the regional cultural and linguistic system are characterized by heterogeneity. The various groups also share a great deal of material and ritual culture as a result of their interaction, as well as a number of grammatical features in their languages. Sociolinguistic studies of the Vaupés and Upper Río Negro areas (e.g. [28,29]) and the nearby Caquetá-Putumayo area (e.g. [30]) have described language ideologies that discourage lexical borrowing, confining the effects of contact predominantly to grammar, which is less consciously manipulable. The outcome of this structured interaction, both in the linguistic and cultural domains, is a combination of assimilation and differentiation in various parts of the systems. Similar situations, for instance cases of low lexical borrowing combined with substantial grammatical diffusion, which Epps associates with consciously maintained differences between groups, are also found in other places in Amazonia, such as the Guaporé-Mamoré area in east Bolivia and west Rondônia, the Upper Xingu, and, bordering Amazonia in the Chaco area and the southern Guyanas [31,32].

Epps hypothesizes that the pattern of social differentiation and complementarity found in the Upper Rio Negro represents a system of sharing geographical space which was more widespread in pre-Columbian Amazonia. In such a system, individual ethnolinguistic groups are associated with particular identity markers (including some linguistic features, and trade specialization), but also take a place within a regional context of interaction. In Epps' words [11, p. 285]: ‘Language plays an essential role as a marker of identity within these regional systems, and local linguistic practices are closely associated with the maintenance and even cultivation of differences.’ One could regard this as a two-tiered ideology, consisting first of a generalized and loose ‘Amazonian package’ based on ‘shared, mutually imbricated understandings to the effect that human bodies are fabricated socially, that this occurs in the context of a perspectival cosmos, and that relations with dangerous outside others are indispensable to this process' ([33, p. 477] as well as references therein). The second tier describes local and regional modalities of such relations with others (e.g. exogamy, trade and shared cultural events), and the relevant identity markers within these local systems of complementarity and interaction (see also [34]).

In short, in a system like this, the persistence of linguistic diversity is an outcome of interaction and contact rather than isolation. This model would predict that high genealogical diversity would persist even in the context of regional integration, without any clear distinction between isolates and larger language families. Subsistence strategies and language expansions that are confined within the Amazon would have had little impact on the systems of interaction. These societies would just be incorporated into the regional system, as observed, for instance, in the Vaupes, where subsistence strategies seem to simply be one of the identity markers [11,28,34].

In what follows, we examine the linguistic diversity of the Northwest Amazon (NWA) in light of the isolation and integration hypotheses outlined above. The NWA includes the Upper Rio Negro and the Vaupes regions described above, and it is home to small language families, isolates and larger family expansions. We test predictions that the isolation and integration hypotheses described above would make for signals based on datasets from four different disciplines: biogeography, cultural anthropology, population genetics and linguistics. We discuss each type of data in turn in §3 (approach) and 4 (results). Before presenting our data, methods and analysis, we briefly introduce the NWA in §2, as well as three (near-)isolate languages which we examine in greater detail.

## 2. The Northwest Amazon

For the purposes of this article, we define the NWA as the area delimited by the Andean mountain range to the west, the northern cordillera in Venezuela to the north and the edge of the Brazilian shield, between the Rio Negro and the Orinoco, in the east (see [35], p. 168]). There is no obvious environmental or ecological border to the south, so we place it at the Marañon River. This is a relatively inclusive interpretation of the northwestern portion of the Amazon, since it includes the eastern Andean slopes and highlands, but this area captures a patchwork of small language families and larger family extensions that is representative of the continental pattern. It includes a number of isolates (e.g. Cofán, Urarina, Puinave and Kamsá), near-isolates (families with two members, e.g. Tikuna-Yuri, Peba-Yaguan, Kakua-Nukak, Cahuapanan and Boran), small language families with under 10 members (e.g. Chicham, Zaparoan, Nadahup and Witotoan), as well as representatives of larger families (Tukanoan, Arawakan, Tupian, Quechuan, Panoan and Cariban). As such, the area is a prime example of one of Diamond's surviving diversity islands, with languages from the full range of family sizes.

In the remainder of this paper, we focus especially on the group of isolates and small language families, to the extent to which we have been able to collect data for these groups. We have assembled a basic sample that is representative of the diversity patterns of the area. This sample of language groups and some attributes of the languages are given in the electronic supplementary material, section S1. In order to obtain a more detailed perspective, and to be able to achieve a systematic point of comparison, we zoom in on three (near-)isolates of the area. These are as follows:

**Kamsá** (Camsá, Kamentsa)^9^ [36] is an isolate language spoken by fewer than 500 people in southern Colombia, predominantly on a plateau at 2000 m.a.s.l., in a passageway between the highlands and lowlands. They have shared this area with the Inga (speakers of a Quechuan language) since around the fifteenth century. In the past, this involved two-way bilingualism. Today, Spanish is being adopted as a lingua franca, and the degree of Kamsá-Inga bilingualism is declining. Kamsá has borrowed Inga and Spanish words into its lexicon.
**Tikuna** (Ticuna) [37] belongs to a small family of two members, along with Yuri, an extinct language for which we have very little linguistic data.^10^ Tikuna is a relatively large language with possibly over 50 000 speakers and close to 70 000 people identifying as Tikuna. The language covers a large territory in northeastern Peru, southern Colombia and northwestern Brazil. Contact effects in the form of borrowed lexicon can be found as a result of contact with Old Omagua and (varieties of) Língua Geral Amazônica (both Tupian), and—to a lesser extent—a variety of Quechuan, and later Spanish and Portuguese.
**Puinave** (Wãnsöjöt) [40] is an isolate language spoken in eastern Colombia, in a transitional zone between the lowland rainforest and the eastern Colombian plains. The Puinave are surrounded by Arawakan-speaking groups. They practice exogamy among language-internal clans, but they also marry speakers of Arawakan languages in the vicinity (Curripaco, Baniwa and Piapoco). There are suggestions in the ethnohistorical literature that the Puinave moved from the Marañon area to their current location. There are also suggestions of links to the Makú, although these seem to lack firm evidence [40].

## 3. Approaches and datasets

### 3.1. Geography

The isolation hypothesis predicts that isolates and smaller languages survive because they have not been replaced by expanding families. These smaller language families and isolates are therefore predicted to survive in niches that are less suitable for agriculture, allowing these groups to persist in relative isolation. From the perspective of geography, this would lead us to expect that the languages belonging to the larger families would, on average, occupy territories that are better suited for agriculture. According to the integration hypothesis, by contrast, geography would be a poor predictor of the size of a family that a particular language belongs to.

To represent language locations, we used point data from the online database Glottolog [7] and used the glottospace function in the glottospace R package [41] to interpolate language locations for the entire South American continent. The resulting polygons were grouped to the level of language families (including isolates) and used as input for a grid-based approach to quantify linguistic endemism—a measure of the geographical uniqueness (i.e. range-restrictedness) of languages or language families in a given area [42]. From the resulting hexagon grid, we selected the grid cells that contained at least one ethnolinguistic group of our sample of 36 languages. To gain a better understanding of these ethnolinguistic groups in their wider geographical context, we expanded our grid by including the 36 surrounding grid cells (i.e. three rings in a hexagon grid surrounding each focal cell). This resulted in a continuous grid that contained all languages of our sample, as well as their intermediate and surrounding areas. While this continuous grid seemed most appropriate, we also assessed the robustness of our findings for smaller and larger grids (*k* = 6,18 and 72, i.e. surrounding grid cells). To assess whether environmental and societal factors play a role in shaping patterns of endemism, we collected a suite of covariates (table 1). These variables were extracted and aggregated for each grid cell of 10 000 km^2^. Range sizes of a language family might not only be affected by environmental conditions, they might also be influenced by neighbouring language families. To assess whether endemism in a given grid cell is influenced by adjacent cells, we fitted a spatial lag model using all environmental predictors and a spatial autoregressive parameter [52]. The performance of this spatial model was compared against the full ordinary least-squares regression model (without a spatial autoregressive parameter) based on Akaike information criterion and using Lagrange multiplier tests as implemented in the spdep R package [52].

**Table 1.** Environmental and climatic covariates of endemism tested in this paper.

no.	variable	type	aggregation	source
1	annual temperature	climate	mean	[43]
2	temperature seasonality	climate	mean	[43]
3	annual precipitation	climate	mean	[43]
4	precipitation seasonality	climate	mean	[43]
5	elevation	topography	mean	[44]
6	roughness	topography	mean	[45]
7	soil constraints	agriculture	mode	[46]
8	crop suitability	agriculture	mean	[47]
9	travel time to cities	societal	mean	[48]
10	travel time to ports	societal	mean	[48]
11	ecoregions	biodiversity	sum	[49]
12	river length	hydrography	sum	[50]
13	population density (2000 AD)	population	mean	[51]
14	population density (1500 AD)	population	mean	[51]

### 3.2. Cultural anthropology

We can also consider the *isolation hypothesis* and the *integration hypothesis* in the light of the patterns of sociocultural diversity attested in the NWA. In particular, if the region's linguistic isolates and small language families are indeed the marginalized remnants of an initial period of population and diversification, a panorama that was then partially disrupted by the more recent expansion of the major Amazonian language families, then we would expect the speakers of those isolates and small language families to engage in social and cultural practices that are notably distinct both from those of the speakers of the larger language families, and from each other. Since the isolation hypothesis is primarily a story about subsistence and economy, we might expect such a distinction in that domain of cultural practices. By contrast, for the integration hypothesis, we might expect that cultural practices, cultural materials and subsistence practices are not bound to specific ethnolinguistic groups and are instead shared by other groups as a result of sustained contact—even as some cultural practices remain distinct, in the context of a system of regional complementary (see discussion above).

To explore this question, we drew on a large database of ethnographic information that was developed for the SAPPHIRE project, based at Leiden University. The database encodes a broad range of variables regarding subsistence activities, trade, food preparation, material culture, the gendered division of labour, settlement types, house building, kinship, marriage practices, social organization, body modification, cosmology, ritual and other domains of sociocultural information. A more detailed description of the database can be found in the electronic supplementary material.

For this paper, we draw from the same sample of ethnolinguistic groups as the genetics and linguistics datasets. However, because ethnographic information is sparse for some of these groups, we excluded groups for which insufficient data are available. Then, we further distinguished the ethnolinguistic groups that belong to large language families (10 or more languages) and to small language families and isolates (fewer than 10 languages). The rationale behind this division is that the smaller language families would represent Nettle's remnants of the initial colonization, while the larger families would represent later spreads into the area.

The next steps in the workflow are identical for the sociocultural and linguistic datasets, so they will be described here and not repeated in §3.4 about linguistics. The first step was to standardize the databases using the glottospace R package [41]. The standardized data format allows us to measure the degree of (dis)similarity between ethnolinguistic groups for both datasets. These distances were calculated using Gower's general coefficient of similarity [53]. The Gower's distances and resulting distance matrices were used as input for non-metric multi-dimensional scaling (NMDS) [54]. NMDS results were subsequently plotted in two and three dimensions which allowed us to explore the degree of dissimilarity between groups. To assess whether pre-defined sets of groups (in this case small and large families) are significantly different from each other, we performed overall and pairwise PERMANOVA on the raw distance matrices [55].^11^

### 3.3. Genetics

In the most extreme interpretation, the isolation hypothesis would predict little to no intermarriage and hence genetic admixture among the small language families or between the small and large language families. By contrast, the integration hypothesis would predict abundant genetic admixture among geographical neighbours, independent of their ethnolinguistic affiliation. To evaluate these contrasting scenarios, we analysed new genome-wide SNP data, generated on the Affymetrix Human Origins Array, from the three linguistic (near-)isolates discussed above (Puinave, Kamsa and Tikuna) and their geographical neighbours. We used these data to distinguish signals of shared evolutionary history and/or genetic admixture from signals due to genetic isolation. Furthermore, we focused on allele-frequency-based approaches that allow us to make inferences about old and recent demographic events, and haplotype-based approaches for which inferences on more recent temporal scales can be made. For the allele-frequency-based approach, we used outgroup-f3-statistics (see [60] for discussion) of the form f_3_(isolate, NWA_groups; Outgroup). In brief, this test measures the shared branch lengths (or shared drift) between each isolate and other NWA_groups in comparison to an outgroup that has diverged long ago from both groups and that has not recently admixed with any of them. Thus, higher f3 values indicate closer genetic relationships between the isolate and the tested NWA group. As an outgroup we used published data from Mbuti individuals [61], a group of foragers living in the Central African rainforest. Furthermore, we used an f4-statistic of the form f_4_ (Neighbour, Native_American; Isolate, Mbuti) to test whether each isolate shares significantly more drift with its closest geographical neighbours than with other Native American groups used as a comparison (electronic supplementary material, figure S4a–c). If the isolate shares more drift with its neighbour than with other geographically distant groups that would result in an f_4_ value that is significantly bigger than zero. By contrast, if the isolate shares more drift with other groups than with its neighbours, it would result in a significantly smaller than zero f_4_ value.

The haplotype-based approach analyses the sharing of long genomic regions between pairs of individuals that are identical-by-descent (IBD), i.e. continuous segments of the genome inherited from a set of common ancestors without recombination [62]. The length of shared IBD blocks is informative about the demographic history of a population going back tens to a few hundred generations before the present [63].

To estimate IBD, we carried out statistical phasing with the software SHAPEIT version 2.r904 [64], using an American reference panel (i.e. Colombians in Medellín, Peruvians in Lima, Puerto Ricans in Puerto Rico and individuals with Mexican Ancestry in Los Angeles) and a recombination map, both from the 1000 Genomes Project Phase 3 (1000 Genomes Project Consortium *et al*. 2015). We ran SHAPEIT with options –burn 10, –prune 10 and –main 30 for iteration number with 500 conditioning states, leaving other parameters as default [65]. We then used the phased output to detect IBD blocks within individuals (homozygous-by-descent or HBD) and between individuals (IBD) with the software RefinedIBD [66], both IBD and HBD were merged and split by length category into three datasets as follows: 1–5 centimorgans (cM), 5–10 cM and over 10 cM, as previously described [65,67]. These datasets were used to quantify the IBD sharing of each linguistic isolate with the other ethnolinguistic groups from NWA. Ralph & Coop [62] have proposed that these length categories are informative about demographic events on the time intervals of 1500–2500 years ago, 500–1500 years ago and 0–500 years ago, respectively.

### 3.4. Linguistics

The isolation hypothesis predicts divergent patterns in grammar, while the integration hypothesis predicts convergent patterns, especially in more abstract grammatical patterns. We approach the issue of isolation versus integration through linguistic distances (in the same manner as the sociocultural data, as described in §3.2). To this end, we developed a database in which the languages of the sample are scored for 73 structural features. The features cover a broad range of grammar, ranging from phonology to syntax, and are easily connected to global distribution data available in [68] or [69], allowing for comparisons of regional NWA patterns to global patterns. For a more detailed description of the variables in the linguistic database, as well as for the language sample, see the electronic supplementary material.

Since the linguistic data come from published grammatical descriptions, we were limited to the languages for which sufficient published material was available. This left us with 36 languages. For more information on the precise sample, see the electronic supplementary material. As in the cultural–anthropological approach in §3.2, we divided the sample languages into those that belong to small language families (fewer than 10 languages) and languages that belong to large language families (10 or more). The isolation hypothesis would predict that the smaller language families, having retracted to or survived in areas where the expansions did not reach, would show significant differences from the languages of the expanding groups. The integration hypothesis predicts that the groups expanding into the NWA engaged in extensive interactions with their new neighbours, leading to linguistic convergence.

We furthermore used visualization techniques (heat-map and correlation plots) to explore the data for genealogical and areal signals. In these visualizations, the isolation hypothesis would predict strong genealogical signals and weak areal signals, while the integration hypothesis would predict strong areal signals, perhaps diffusing the genealogical signal.

## 4. Results

### 4.1. Geography

**Figure 2.**
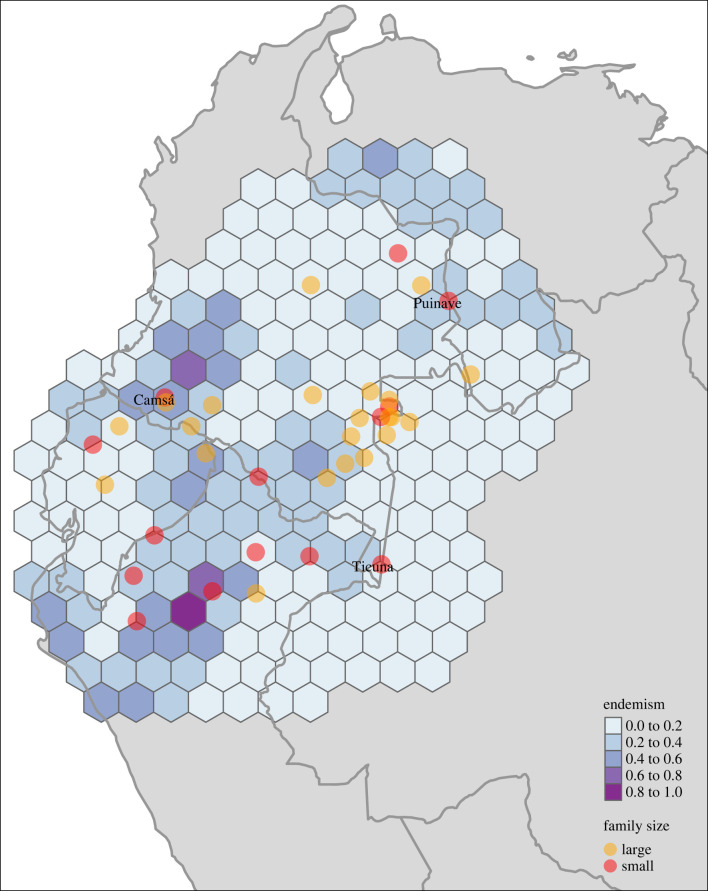
Linguistic endemism in the Northwest Amazon. Each cell has an area of 10 000 km^2^. The languages of special focus are plotted for reference.

Our grid-based calculation of linguistic endemism in the NWA indicates that narrow-ranged language families tend to cluster in particular geographical areas (figure 2). Linguistic endemism is particularly high in northwestern Peru and southern Colombia, and to a lesser extent in the southwestern parts of Venezuela. The three ethnolinguistic groups that are the focus of this study, Kamsá, Puinave and Tikuna are located in areas of varying degrees of endemism. Kamsá is surrounded by areas of intermediate and high endemism, suggesting the area is characterized by several narrow-ranged language families. While linguistic endemism in the Puinave area is lower than for Kamsá, endemism values surrounding Puinave are slightly higher than their further neighbours. While Kamsá and Puinave are both within ‘islands’ of elevated endemism, this is not the case for Tikuna, which is rather at the boundary of areas characterized by more widespread language families.

The Lagrange multiplier tests for error dependence and a missing spatially lagged-dependent variable were both significant. Of the robust versions of these tests, only the spatial lag statistic was significant and therefore we report here the results of the spatial lag model. Overall, the spatial lag model performed better than the linear model (ΔAIC: 97). The spatial autoregressive parameter had a positive value and was highly significant (*p* < 2.22 × 10^−16^), indicating that endemism in a given area tends to increase with increasing endemism in surrounding grid cells (independent of the other parameters in the model). Centres of linguistic endemism (concentrations of narrow-ranged language families) in the NWA are directly related to precipitation, travel time to urban centres and ecological diversity. The concentration of narrow-ranged families tends to decrease with annual precipitation and, to a lesser extent, precipitation variability. Stated differently, large-ranged language families in the study area are more likely to be found in humid areas (and areas with variable rainfall). Additionally, there was a negative effect of travel time to cities on endemism (direct effects in table 2; for a study area of 36 cells surrounding each focal cell). Finally, in those areas with a larger number of ecoregions, endemism increases as well, suggesting that ecologically heterogeneous areas in the NWA house a large number of range-restricted language families.

**Table 2.** Impacts of spatial lag model for focal cells and 36 surrounding cells. Significance levels: n.s. = *p* > 0.1; . = *p* ≤ 0.1; * = *p* ≤ 0.05; ** = *p* ≤ 0.01; *** = *p* ≤ 0.001. Predictors are scaled.

variable	direct	indirect	total
annual temperature	0.042	0.1	0.142
temperature seasonality	0.16	0.385	0.545
annual precipitation	−0.212*	−0.51 .	−0.722*
precipitation seasonality	−0.2 .	−0.482	−0.682
elevation	−0.066	−0.16	−0.226
roughness	−0.003	−0.006	−0.009
soil constraints	−0.058	−0.141	−0.199
travel time (cities)	−0.158 .	−0.381	−0.539 .
travel time (ports)	−0.017	−0.04	−0.056
ecoregions	0.128*	0.307 .	0.435 .
river length	0.09	0.216	0.306
crop suitability	0.004	0.009	0.012
population density (1500 AD)	−0.084	−0.203	−0.288
population density (2000 AD)	0.061	0.148	0.209

To assess whether these results were also valid at smaller and larger extents, we ran the same analyses for *k* = 6,18, and 72 (number of cells surrounding each focal cell; results in the electronic supplementary material). For both the smaller and larger extents, the simple tests for spatial dependence were significant. For the robust tests, the test for error dependence was never significant, while the test for a missing spatial lag generally was (*k* = 6, *p* = 0.078; *k* = 18, *p* = 0.0005637, *k* = 72, 3.146 × 10^−5^). For the smallest extent (*k* = 6), the significant direct parameters (*p* < 0.1) in the spatial model were ecoregion richness and travel time to ports. When including 18 surrounding grid cells, ecoregion richness and river length had a significant direct impact. At the largest extent, only travel time to cities had a significant impact. The direction of the relationship in each of the models did not change. To summarize, at the smaller extents, ecoregion richness had a significant impact on endemism, while this effect was no longer evident at the largest scale.

### 4.2. Cultural anthropology

**Figure 3.**
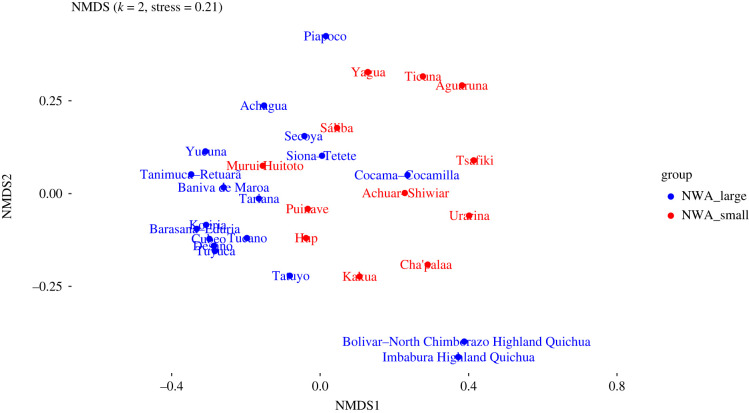
Two-dimensional NMDS plot of sociocultural data (all variables).

An NMDS plot generated from the dataset described in §3.2 is shown in figure 3. Ethnolinguistic groups whose languages belong to large families are shown in blue, and those whose languages belong to small families are shown in red. (Note that according to [70], stress values are most reliable below 0.1, while general conclusions can be drawn between 0.1 and 0.2, and values above 0.2 can be misleading. Therefore, we have relied on raw distances matrices and 3D plots for exploration.)

Here, we can discern a general difference between the cultural practices of people who speak languages from large families (blue), and those who speak languages from small families (red). The former group is fairly broadly distributed to the left of this NMDS plot, clustering densely in some places, while the latter group is more confined to the right and centre area, but shows a fair degree of internal heterogeneity. To test these impressions, we carried out a PERMANOVA test (see §3.2) to ascertain whether the centroids of the small families and large families are significantly different (table 3). We found that the difference is indeed statistically significant (*p* = 0.003).

**Table 3.** Results of the PERMANOVA test comparing languages belonging to small families with languages belonging to large families on the basis of cultural data. *** = *p* ≤ 0.001; ** = *p* ≤ 0.01; * = *p* ≤ 0.05; n.s. = *p* > 0.05.

group 1	group 2	*p*-value (adj)	sign (adj)
NWA_large	NWA_small	0.003	**

While these patterns are clear, it is important to point out that, by the nature of the analysis itself, many of the languages in the blue group (members of large language families) are related to each other genealogically, and thus that their speakers can be expected to share some common history. Furthermore, some of these groups are geographically close to each other (as speakers of related languages often are).

Table 4 shows a more nuanced pattern. Here we separated out the speakers of languages from the Tukanoan and Arawakan families, which have undergone a long history of intensive interaction, as has been described thoroughly in the anthropological and ethnohistorical literature (e.g. [71]). This is not the case for connections between either language family and the smaller families of the area (individual exceptions notwithstanding). The rest of the languages from large families, now reduced to two Quechuan languages and Kokama-Kokamilla (Tupian), show no significant difference from either the small language families or Arawakan languages.^12^ We can tentatively conclude from this that there seems to have been a special, shared socio-historical dynamic linking the expanding Arawakan and Tukanoan groups on the one hand, and a separate dynamic involving the smaller families (and some of the larger families) on the other.

**Table 4.** Results of the PERMANOVA test comparing sociocultural practices corresponding to languages from small families with those corresponding to languages from large families, on the basis of a sociocultural dataset, separating out Tukanoan and Arawakan languages, and grouping the remaining languages of large families in a reduced large language family group. *** = *p* ≤ 0.001; ** = *p* ≤ 0.01; * = *p* ≤ 0.05; n.s. = *p* > 0.05.

group 1	group 2	*p*-value (adj)	sign (adj)
Arawakan	Tukanoan	0.912	n.s.
Arawakan	NWA_small	0.03	*
Arawakan	NWA_large (reduced)	0.078	n.s.
Tukanoan	NWA_small	0.006	**
Tukanoan	NWA_large (reduced)	0.036	*
NWA_small	NWA_large (reduced)	0.144	n.s.

**Figure 4.**
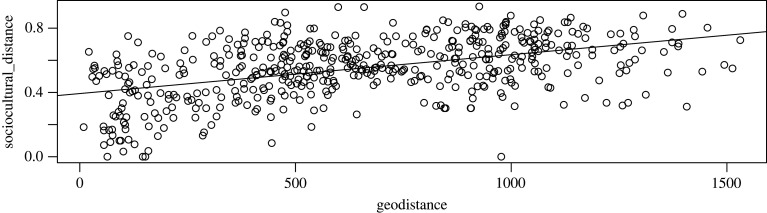
Correlation plot of sociocultural (*y*-axis) versus geographical (*x*-axis) distance (entire sample).

**Figure 5.**
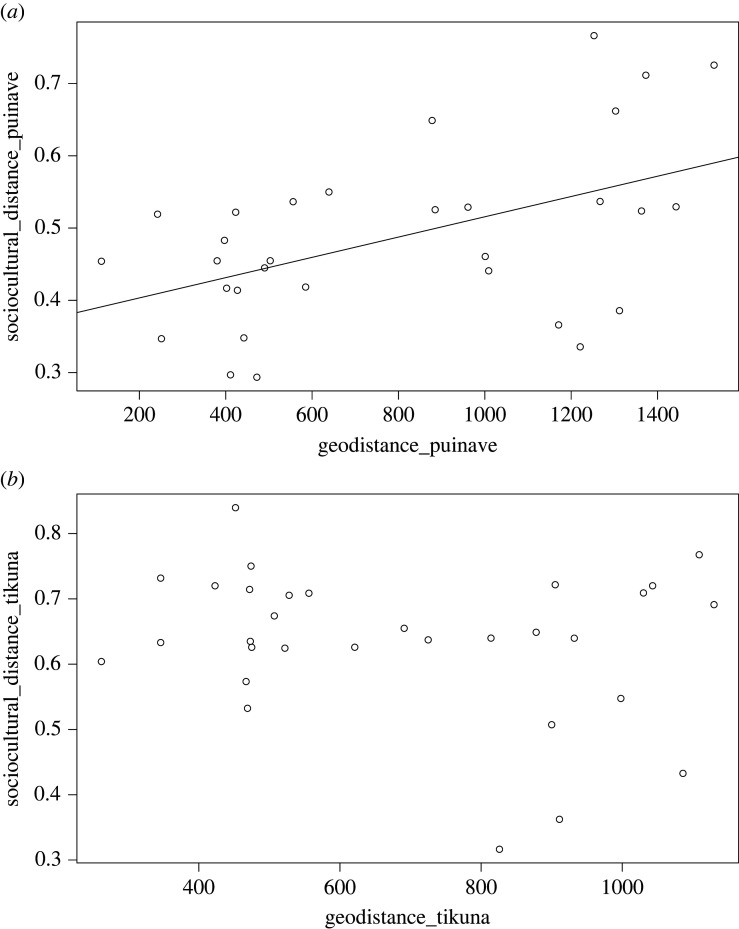
(*a*) Correlation plot of sociocultural distance versus geographical distance for Puinave. (*b*) Correlation plot of sociocultural distance versus geographical distance for Tikuna.

In order to assess areal effects, we correlated geographical distance and cultural distance, which provide us with a general trend of the correlation between sociocultural distance and geographical distance, shown in figure 4. The trend indeed seems to be that, on average, groups are similar to their geographical neighbours. However, if we zoom in on two of the three focal groups introduced above (Puinave and Tikuna—for Kamsá there are not enough data), we see that this trend is not universal (figure 5): whereas Puinave (figure 5*a*) seems to be most similar to its closer neighbours, the opposite is true for Tikuna (figure 5*b*). We can conclude from this that, although there is certainly a trend to exchange ideas and practices with the groups in the geographical vicinity, this is not true for all groups, so that there does not seem to be a generally applicable narrative for the NWA.

For the purposes of this paper's goals, it is also relevant to look more closely at the sociocultural variables related to subsistence and economy. This is because Nettle's proposal identifies changes in subsistence practices as part of the second punctuation, as languages associated with agriculture would have expanded and marginalized their neighbours. If the scenario is correct, we might expect to find differences in subsistence and economic practices between speakers of languages from large (i.e. expansive) and small South American families. The proposal of Epps, on the other hand, does not identify subsistence and economy as a relevant consideration, since most of the groups in the NWA practice agriculture.

**Figure 6.**
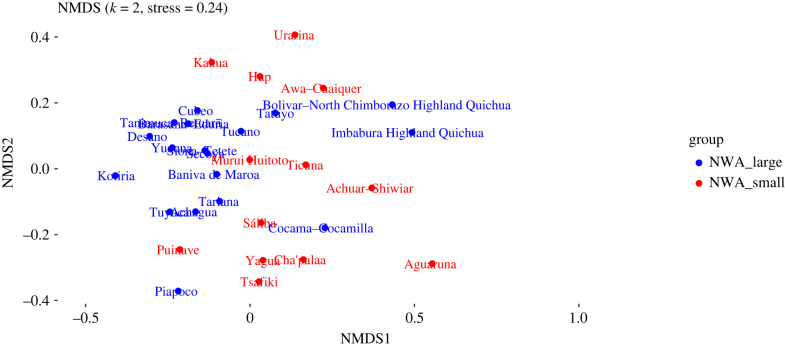
Two-dimensional NMDS plot cultural data, economy-related variables only.

We subset the dataset described above to only the variables relevant to subsistence and economy, broadly construed to include tools and techniques for hunting, fishing, gathering, and all manner of crop production, processing and consumption (including both food and ceremonial crops); trade and transportation; and material culture such as weaving, clothing, ceramics and woodworking. An NMDS plot generated from the data is shown in figure 6.

Here, we can see a similar pattern to the plot in figure 3 above: speakers of languages from large families are generally found together to the left (blue), while speakers of languages from small families and isolates are more widely distributed and less coherent (red) to the extent that they differ from each other significantly.

For the PERMANOVA results, we again first looked at the difference between small and large language families (table 5). Again, there is a significant difference between small and large language families.

**Table 5.** Results of the PERMANOVA test comparing economy-related sociocultural practices corresponding to languages from small families with those corresponding to languages from large families, on the basis of the sociocultural dataset. *** = *p* ≤ 0.001; ** = *p* ≤ 0.01; * = *p* ≤ 0.05; n.s. = *p* > 0.05.

group 1	group 2	*p*-value (adj)	sign (adj)
NWA_large	NWA_small	0.016	*

To get a better view of the more specific patterns, we split up Tukanoan and Arawakan, yielding the values in table 6.

**Table 6.** Results of the PERMANOVA test comparing economy-related sociocultural practices corresponding to languages from small families with those corresponding to languages from large families, on the basis of the sociocultural dataset, separating out Tukanoan and Arawakan languages. *** = *p* ≤ 0.001; ** = *p* ≤ 0.01; * = *p* ≤ 0.05; n.s. = *p* > 0.05.

group 1	group 2	*p*-value (adj)	sign (adj)
Arawakan	Tukanoan	1	n.s.
Arawakan	NWA_small	0.198	n.s.
Arawakan	NWA_large (reduced)	0.06	n.s.
Tukanoan	NWA_small	0.018	*
Tukanoan	NWA_large (reduced)	0.018	*
NWA_small	NWA_large (reduced)	1	n.s.

From these results, we can conclude that there are common subsistence patterns in the area, that Arawakan is fully integrated into this pattern, and that Tukanoan stands somewhat apart.

Our interpretation of this pattern is consistent with the integration hypothesis of Epps, in the sense that all of the groups in the sample practice agriculture to some degree. A possible further interpretation is that Arawakan groups played an important role in spreading subsistence strategies without incorporating the smaller families linguistically; this would explain why Arawakan speakers are so similar in this respect to their neighbours from a range of small language families. Nevertheless, there is some amount of meaningful difference between the economy-related cultural practices of the Tukanoan groups and the language families with a smaller representation in the area (whether large or small families). The areal signals for the economy-related variables are similar to those of the full dataset. Correlation plots are shown in section S2.2 of the electronic supplementary material.

By way of conclusion, we can say that, although there are some significant differences between the cultural practices associated with languages from small families and those associated with languages from larger families, this is less clear in the subsistence strategies, where Arawakan-speaking groups in particular are culturally similar to both the Tukanoan-speaking groups and the speakers from smaller families. Tukanoan interactions, on the other hand, seem to have been mostly with Arawakan groups. All in all, then, there is some evidence of independent developments of larger and smaller language families, in line with the isolation hypothesis, but this does not appear to hold entirely for subsistence strategies, where the role of Arawakan seems to be more in line with the integration hypothesis.

### 4.3. Genetics

**Figure 7.**
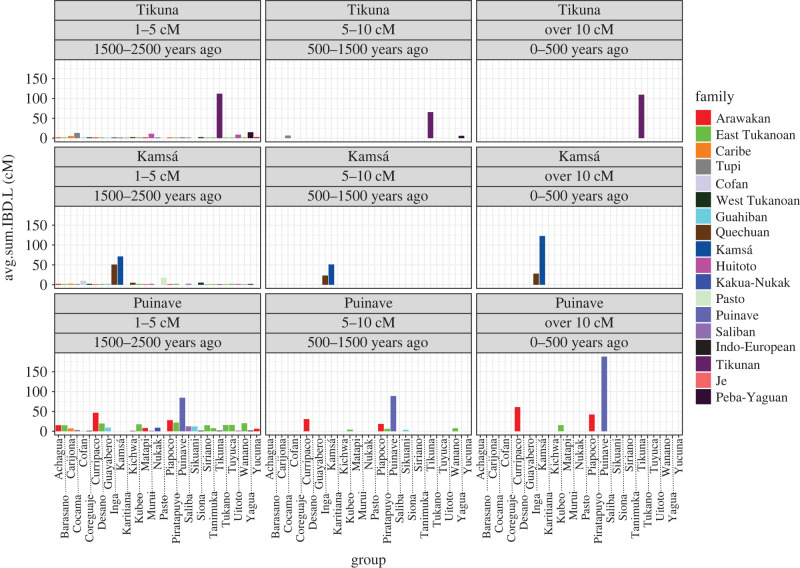
Patterns of IBD sharing between each of the three (near-)isolates and other groups from NWA. Length categories 1–5 cM, 5–10 cM and greater than 10 cM are informative about demographic events on the time intervals of 1500–2500 years ago, 500–1500 years ago and 0–500 years ago, respectively.

The results of the IBD block sharing analysis between the (near-)isolates and NWA groups appear in figure 7, the comparisons for the whole genetic dataset^13^ appear in the electronic supplementary material, figure S3. We divided IBDs into three block lengths, roughly corresponding to time periods of 2500–1500 years ago, 1500–500 years ago and 500 years ago to the present, respectively. Tikuna consistently shows limited IBD sharing through time with other NWA groups and during the last 500 years IBD sharing is restricted within the group, not even with their closest neighbours Cocama and Yagua, hence marriages are likely only or mainly within the group. In the period prior to that there is a low level of shared IBD with nearby Yagua and Cocama; while in the period between 2500 and 1500, many groups in NWA exhibit low-level sharing of IBD blocks. It has been shown that, as we go further back into the past, and in populations that have sufficiently mixed, the probability of sharing many genealogical ancestors becomes greater, and the number of expected IBD segments increases, as shorter segment lengths are considered [62,63]. Although this low-level sharing can be interpreted as background common ancestry, we see that Yagua, Cocama and Huitoto speaking groups stand out in this comparison (1–5 cM), which might suggest that during this time period there were more interactions between Tikuna and these groups. A slightly different pattern is found for Kamsá, which shows a high amount of IBD sharing both within, as well as with the neighbouring Inga (Quechuan) across all length categories. Puinave is an example of one of the groups from the lower Orinoco that shows signs of exogamy with the local groups Piapoco and Curripaco (both Arawakan) in the last 500 years. In addition, in the period between 1500 and 500 years before present we also observe IBD sharing with Sikuani (Guahiban), and three Eastern-Tukanoan groups. In contrast with Tikuna and Kamsá, Puinave shows more IBD sharing in the oldest time period (1–5 cM) with several NWA groups, particularly with Arawakan and Eastern-Tukanoan-speaking groups.

Taking a broader areal view, based on the figures in S3 of the electronic supplementary material, a first observation to make is that the two most recent periods show a difference between the Arawakan and East Tukanoan groups on the one hand, and most of the other groups on the other, in that the former two groups show patterns of IBD sharing with several other groups, while the other groups generally restrict themselves to one other group, or none.

A second observation is that the historical dynamics are different across the sample for the earliest time period, 2500–1500 years ago, than the two more recent periods. In the earliest time period, it can be observed that, at the centre of the NWA, groups that live along the Vaupes, tributaries of the Orinoco, and the Miriti-Parana rivers, show considerable amounts of IBD sharing, which indicate common ancestry and perhaps genetic admixture as well during this time frame. This however, cannot be due to very old common ancestry deriving from the initial peopling of South America, since groups outside the NWA (Karitiana and Xavante) do not show a similar pattern (figures in section S3 of the electronic supplementary material). We also observe some geographical patterning in the amount of IBD sharing and this might reflect the differences in sampling efforts across regions within the NWA, a higher number of groups come from the aforementioned area. Which might indicate that individuals from this area share more of their ancestors and our power to detect these common ancestors is influenced by our widespread sampling of the ethnolinguistic diversity from this location (see [62] for discussion).

**Figure 8.**
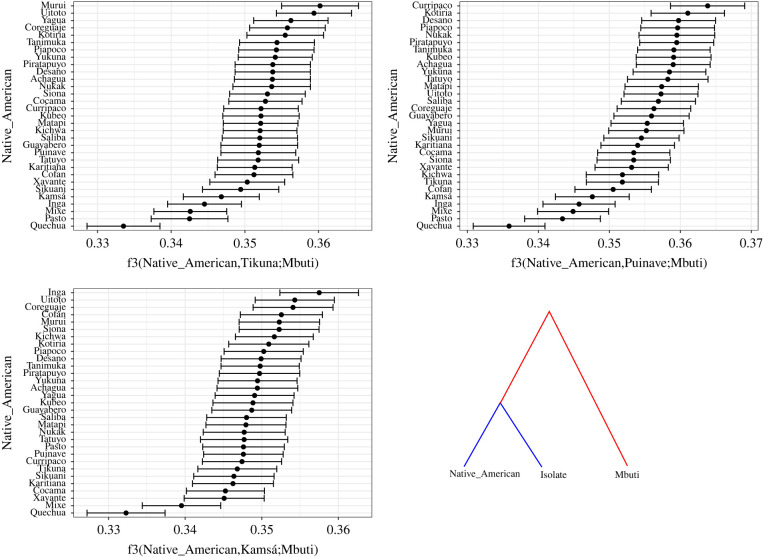
Outgroup-f3-statistic plots for Tikuna, Puinave and Kamsá.

Some of the old signals of shared history between the isolates and other NWA groups are supported by the outgroup-f_3_-statistic (figure 8) and the f4-statistic (electronic supplementary material, figure S4*a*–*c*), which are more informative of older periods (see §3.3 above). In particular, we observe that Tikuna shares more drift with Yagua than with Cocama, its two neighbours. However, Tikuna shares significantly more drift with Murui and Uitoto (electronic supplementary material, figure S4*a*), who live further north along the middle-Putumayo River. In the case of Puinave, the outgroup-f3-statistic confirms the close relationship with Curripaco, its closest geographical neighbour, but also to several other groups in NWA. The same is true for Kamsá, which shows the highest affinities with its geographical neighbour, Inga, but also to other groups from the upper-Putumayo and upper-Caqueta Rivers (figure 8; electronic supplementary material, figure S4*c*).

### 4.4. Linguistics

**Figure 9.**
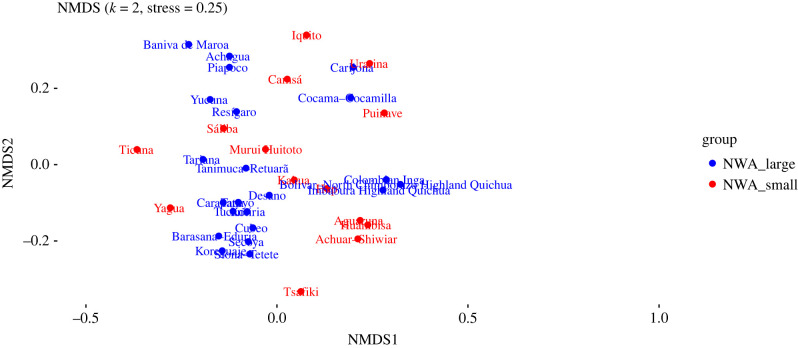
Two-dimensional NMDS plot of linguistic data.

As with the cultural anthropological data, we divided the languages into two groups: those belonging to small families (fewer than 10 members) and those that belong to large families (10 or more). The resulting NMDS plot is given in figure 9, the PERMANOVA results in table 7.

**Table 7.** Results of the PERMANOVA test comparing languages belonging to small families with languages belonging to large families on the basis of linguistic data. *** = *p* ≤ 0.001; ** = *p* ≤ 0.01; * = *p* ≤ 0.05; n.s. = *p* > 0.05.

group 1	group 2	*p*-value (adj)	sign (adj)
NWA_large	NWA_small	0.197	n.s.

**Figure 10.**
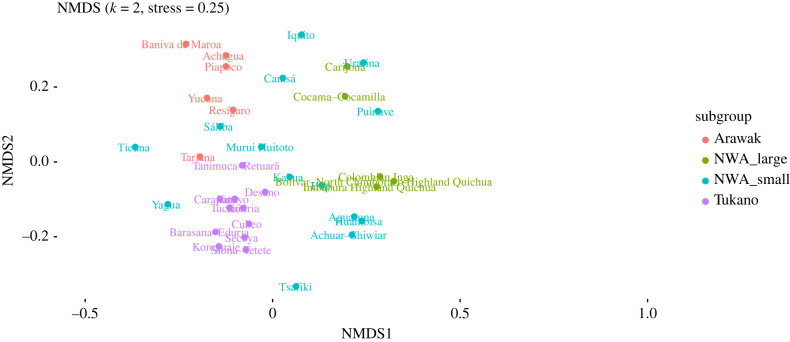
Two-dimensional NMDS plot of linguistic data, coloured by the groups corresponding to table 7: Arawakan, Tukanoan, small language families, and large families except Arawakan and Tukanoan.

Table 7 shows that there is no significant difference between the groups of small-family members and large-family members. Nevertheless, this does not necessarily mean that the languages are similar. In fact, there seems to be a significant amount of genealogical substructure. This becomes clear if we split Tukanoan and Arawakan (table 8), as we did for the cultural anthropological data. The NMDS visualization coloured by the groups in the PERMANOVA table is given in figure 10.

**Table 8.** Results of the PERMANOVA test comparing linguistic data corresponding to languages from small families with those corresponding to languages from large families, on the basis of a linguistic dataset, separating out Tukanoan and Arawakan languages. *** = *p* ≤ 0.001; ** = *p* ≤ 0.01; * = *p* ≤ 0.05; n.s. = *p* > 0.05.

group 1	group 2	*p*-value (adj)	sign (adj)
Arawakan	NWA_small	0.012	*
Arawakan	Tukanoan	0.006	**
Arawakan	NWA_large (reduced)	0.006	**
NWA_small	Tukanoan	0.006	**
NWA_small	NWA_large (reduced)	0.492	n.s.
Tukanoan	NWA_large (reduced)	0.006	**

From these results, a pattern emerges in which the languages that belong to large families do not form a coherent pattern, and in fact all but one group (small versus large—not including Arawakan and Tukanoan) are significantly different from each other. This suggests a strong genealogical signal^14^ and—bar a few local patterns (e.g. the Vaupés languages Tariana, Hup, Kakua and their Tukanoan neighbours)—there seems to be no obvious areal pattern.

A second question to be addressed is whether we can discern a contact signal between the languages of our sample. To this end, we first added a group of control languages, spoken outside the NWA as defined above, and applied another PERMANOVA test, between the groups of small and large NWA families and the control languages. Results are given in table 9.

**Table 9.** Results of the PERMANOVA test comparing linguistic data corresponding to NWA languages from small families with those corresponding to languages from large families, as well as languages from outside the NWA area. *** = *p* ≤ 0.001; ** = *p* ≤ 0.01; * = *p* ≤ 0.05; n.s. = *p* > 0.05.

group 1	group 2	*p*-value (adj)	sign (adj)
NWA_large	NWA_small	0.627	n.s.
NWA_large	control	0.003	**
NWA_small	control	0.024	*

**Figure 11.**
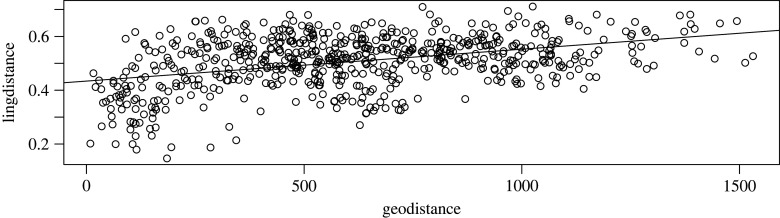
A regression plot of linguistic distance (*y*-axis) and geographical distance (*x*-axis) for all pairs of the sample (excluding control languages).

The fact that both NWA groups are significantly different from the control languages while not from each other suggests some convergence among the NWA languages. This is also suggested by figure 11, which suggests a linear relation between geographical distance and linguistic distance.

**Figure 12.**
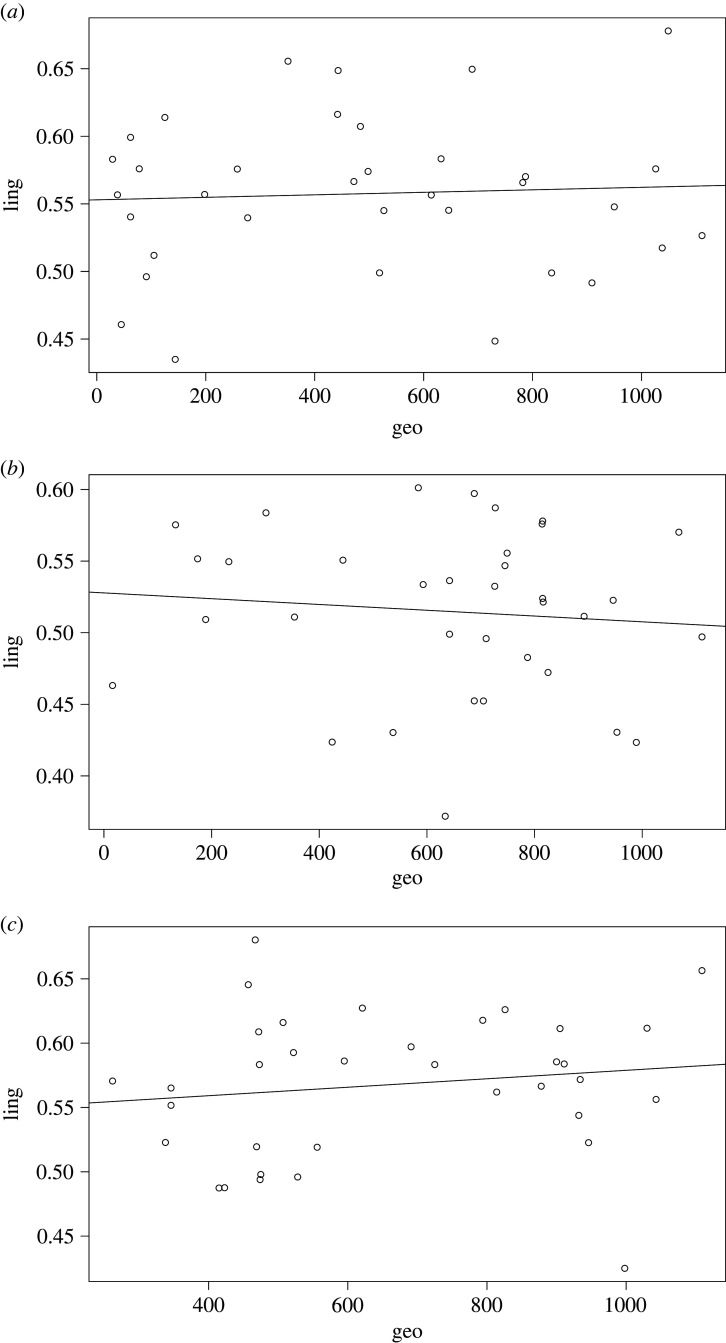
(*a*) Relation between geographical distance and linguistic distance for Puinave, with respect to the other languages in our NWA sample (excluding control languages). (*b*) Relation between geographical distance and linguistic distance for Kamsá, with respect to the other languages in our NWA sample (excluding control languages). (*c*) Relation between geographical distance and linguistic distance for Tikuna, with respect to the other languages in our NWA sample (excluding control languages).

Nevertheless, the correlative pattern does not seem to be universally present in the sample. This becomes clear if we zoom in on our three case studies: Puinave (figure 12*a*), Kamsá (figure 12*b*) and Tikuna (figure 12*c*).

Figure 12*b* shows that, for Kamsá, there is a weak negative correlation between linguistic and geographic distance, suggesting few contact effects resulting from interaction with their neighbours. There is a weak positive relation for Puinave (figure 12*a*) and Tikuna (figure 12*c*). This can be attributed mainly to phonological features (see figures S8 and S9 of the electronic supplementary material), which suggests that there was contact, but likely relatively superficial (see section S4.3 of the electronic supplementary material).

**Figure 13.**
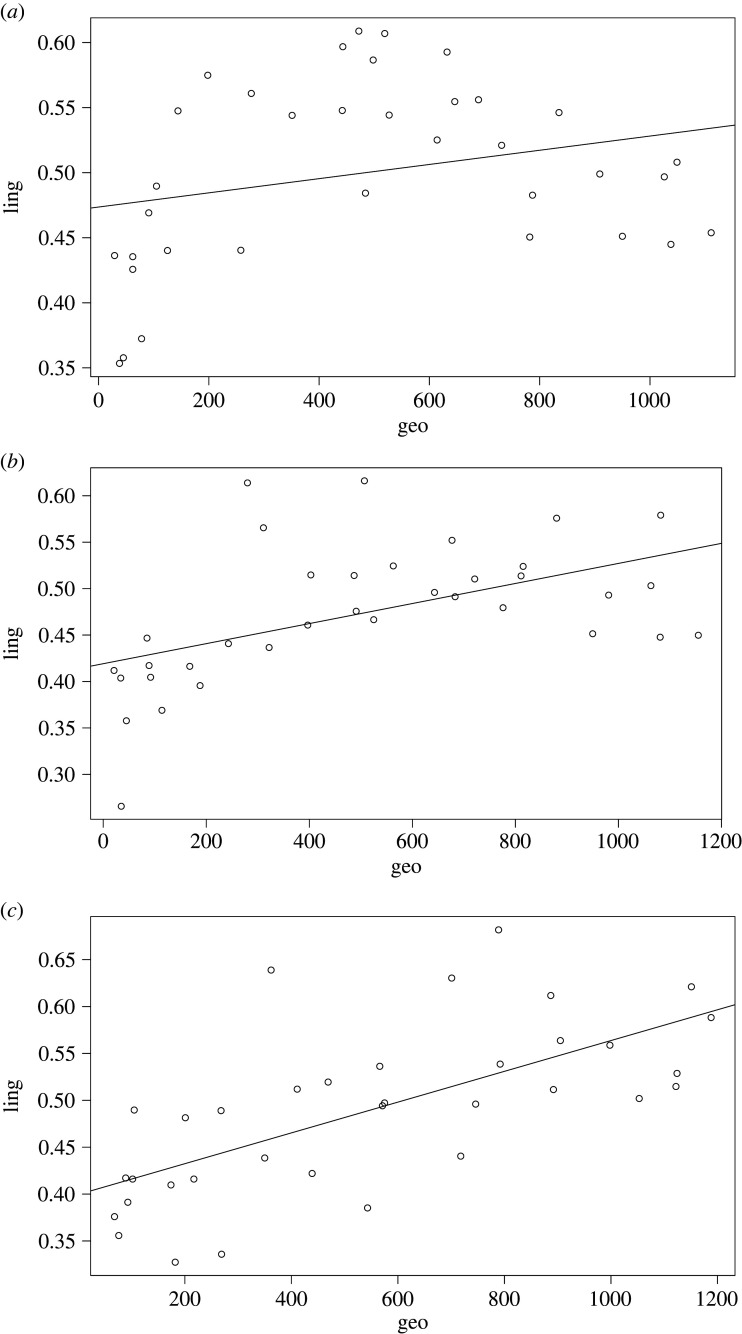
(*a*) Relation between geographical distance and linguistic distance for Hup, with respect to the other languages in our NWA sample (excluding control languages). (*b*) Relation between geographical distance and linguistic distance for Kakua, with respect to the other languages in our NWA sample (excluding control languages). (*c*) Relation between geographical distance and linguistic distance for Tariana, with respect to the other languages in our NWA sample (excluding control languages).

When compared to three other languages from the sample, which form part of the Vaupés area, where we know intensive interactions have taken place for a long time [28,34], we see a different pattern, shown in figure 13*a* (Hup), figure 13*b* (Kakua) and figure 13*c* (Tariana).

**Figure 14.**
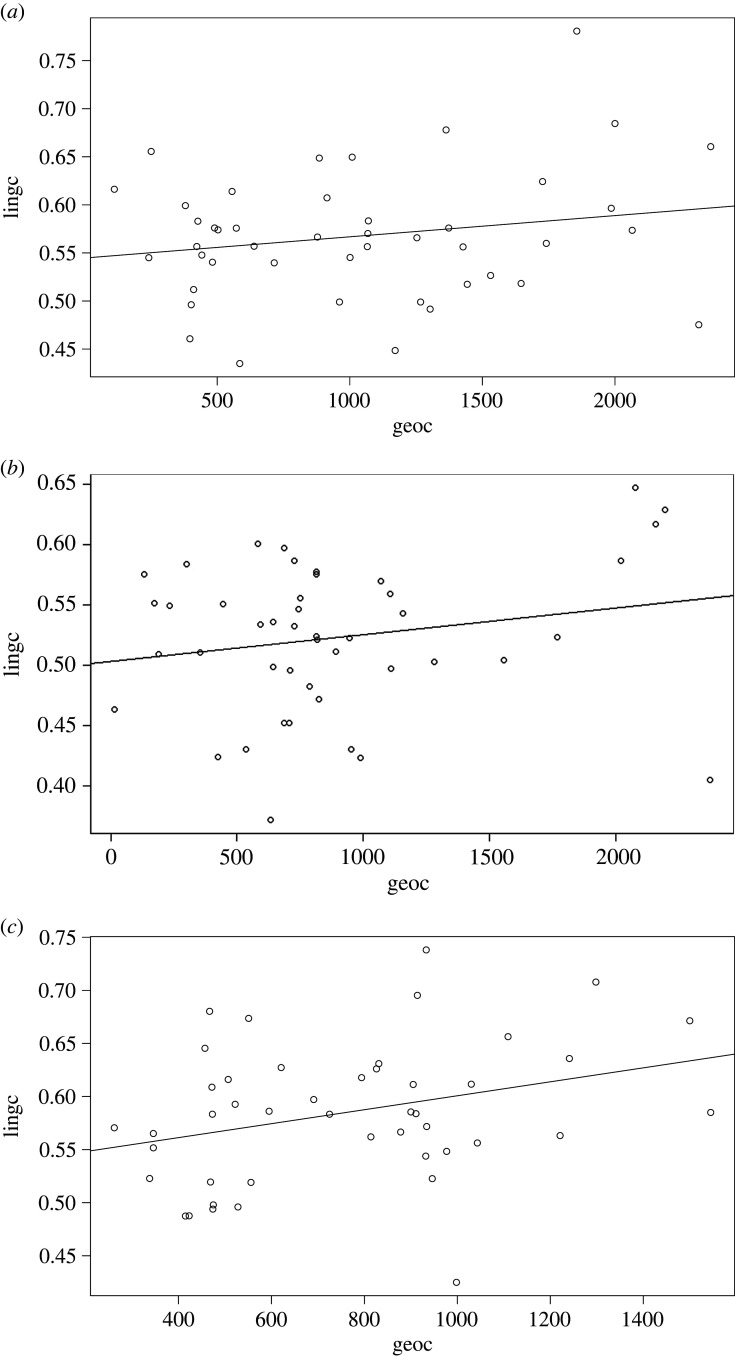
(*a*) Relation between geographical distance and linguistic distance for Puinave, with respect to the other languages in our NWA sample including control languages. (*b*) Relation between geographical distance and linguistic distance for Kamsá, with respect to the other languages in our NWA sample including control languages. (*c*) Relation between geographical distance and linguistic distance for Tikuna, with respect to the other languages in our NWA sample including control languages.

Note that Puinave (figure 14*a*), Kamsá (figure 14*b*) and Tikuna (figure 14*c*) all show geographical proximity effects when the control languages are included.

## 5. Discussion

We started out this paper by contrasting isolation and integration as drivers for the maintenance of linguistic diversity in the NWA. We mainly focused on how smaller language families and isolates may have responded to expanding families as they became increasingly prominent and widespread. We looked at this question from four disciplinary perspectives: biogeography, cultural anthropology, genetics and linguistics. For all four disciplines, we found mixed signals, which in our view suggests that, rather than a single scenario for diversity maintenance in the NWA (let alone for the Amazon or for the Americas), it is more likely that several different scenarios played out in various places and at various times.

To begin the discussion, we summarize the distinction between the isolation and integration hypothesis in table 10.

**Table 10.** Expected signals for the four disciplinary perspectives, geography, anthropology, genetics and linguistics, for each of the diversification hypotheses (isolation versus integration).

	isolation	integration
geography	small families (SF) in isolated areas, less well equipped for agriculture	no areal marginalization of SFs, no significant distribution over ecologies
anthropology	significant differences in cultural profiles as a result of isolated development	local or regional convergence towards common cultural profiles as a result of contact
genetics	little evidence of gene flow between SFs and LFs as a result of intermarriage	abundant evidence of gene flow between SFs and LFs as a result of intermarriage
linguistics	significant differences in linguistic profiles as a result of isolated development	local or regional convergence towards common cultural profiles as a result of contact

All four disciplinary perspectives give mixed signals. Regarding geography, ecoregion richness and annual precipitation had the most significant impact on linguistic endemism. This indicates that those parts of our study area with higher diversity in terms of ecoregions tend to have a high concentration of narrow-ranged language families. This suggests that the NWA's fragmented ecologies have constrained the region's language expansions, and that the smaller families have survived in these ecologically rich areas for that reason. This effect of ecoregion diversity on linguistic endemism was present at all smaller scales, but not when considering the larger region. This could indicate that ecoregion diversity is mainly important at the level of local differences. Although endemism and total language richness are not the same, our findings are in line with an earlier study on North American language richness [71] in which—of all parameters considered—ecoregion richness had the strongest direct effect on language diversity. Similarly, the effect of rainfall on linguistic diversity was also observed in an earlier continent-scale study [72]. These signals are consistent with the isolation hypothesis.

Besides potential socio-environmental drivers of linguistic endemism, we also considered the possible role of endemism in surrounding cells. Across different spatial extents considered here, our findings indicate that a concentration of narrow-ranged families in a given location is positively influenced by the presence of other narrow-ranged families in the surrounding area. This would also be expected under the isolation hypothesis. On the other hand, we found that travel time to cities had a negative effect on linguistic endemism, a finding that goes against the idea that narrow-ranged families survive in refugia further removed from urban centres. This suggests that the smaller language families were not necessarily grouped in remote areas that are difficult to access.

Our analysis of the sociocultural dataset yielded a significant difference between the speakers of languages from small and large families, which on closer inspection can be explained as clear signals of a specific convergence between speakers of Tukanoan and Arawakan languages, and of a difference between both of those large families to the neighbouring smaller families. This result seems to tentatively and partly support the isolation hypothesis, in the sense that the exchange of ideas and borrowing of cultural practices appears to have been more prominent between speakers of Arawakan and Tukanoan languages than between either of those families and the smaller families.

When we zoomed in on variables related to economy and subsistence strategies, we found a pattern that suggested convergence between Arawakan cultural profiles and those of the smaller language families. A closer inspection of the subsistence data reveals that the differences are generally in the kinds of crops that are cultivated—not whether agriculture is present or not—and how those crops are processed and consumed. For instance, speakers of Arawakan and (especially Eastern) Tukanoan languages in the NWA tend to emphasize bitter manioc as a staple crop with a relatively uniform set of processing tools and techniques, while these are more inconsistent among the small families; and coca production and consumption (particularly as a domain of male cultural expertise), which is both less common and less strongly gendered among the smaller families. The same pattern can be seen regarding ayahuasca, the greater presence of weaving among the small families than the large families, and the more consistent presence of canoes among the larger families (which itself is surely due in part to the predominance of the large families along major, navigable rivers). Economic specialization has been described by [11,33] as one of the crucial ingredients of the Amazonian package, which supports identity preservation and exchange. The subsistence data, then, mostly support the integration hypothesis.

With respect to the genetic signals, the last 1500 years show a decrease of gene flow between the societies of the area, except for a number of interactions involving East Tukanoan groups among themselves, and between Arawakan and Tukanoan groups, and to a lesser extent, between Arawakan and local groups in the north along the lower Orinoco. The first millennium AD is associated with an increase of high-intensity landscape management and long-term sedentary stability, population growth and the development of increasingly hierarchical societies in the area [35, pp. 175–184, and references therein]. It is possible that the increasing demographic pressure caused by the intensification of agriculture around the first half of the first millennium AD changed the social dynamics in the area, which may have involved a transformation from groups of roughly equal power to one of increasing inequality, and that ideas about potential marriage partners started to change.

The linguistic signals showed that genealogy seems to have been the main structuring factor in the linguistic profiles of the NWA. In addition, there was some degree of convergence for the entire area, with no significant difference between small and large language families. Zooming in on individual languages suggested differential patterns in different local areas, ranging from clear local convergence, to hardly any convergence. These patterns suggest three things, in our view:

1. Family profiles tend to be robust, suggesting either strong maintenance tendencies or a lack of contact (consistent with the isolation hypothesis).
2. The large-scale areal convergence does signal contact (consistent with the integration hypothesis), but not necessarily local. This is possibly due to high mobility of the different groups, and/or a geographically wide-ranging exchange system [74,75].
3. The patterns of individual language signal differential communicative relations among speakers of individual languages and their neighbours, contradicting any hypotheses that spell out a single story for the NWA.^15^

We interpret the mixed signals discussed above as suggestive of different phases in the socio-historical dynamics of the area. We can tentatively propose two phases as in table 11.

**Table 11.** Suggested interpretation of the signals in terms of historical phases.

phase	description	signals
I (pre *ca* 500 AD)	shared history, which may point to common ancestry or admixture	genetics: signals of shared history throughout the area, with some differences in extent between the groups. Shared history postdates the initial peopling of South America, because control languages show no such signals linguistics: linguistic commonalities throughout the area
II (post *ca* 500 AD)	intensive agriculture, demographic growth, expansions, sedentism, intensive interactions East Tukanoan and Arawakan, generally less intensive interactions smaller language families	geography: smaller families pushed towards areas less equipped for agriculture anthropology: differential cultural profiles, but with some convergence in subsistence strategies, possibly driven by Arawakan groups linguistics: weak local areal signals (with exceptions), stronger genealogical signals genetics: contact and admixture between expanding language families and small families in some areas, in particular among East Tukanoans and between East Tukanoans and Arawakans; decreased gene flow among smaller groups in some areas, accelerated in most recent times as a consequence of European arrival

We can possibly distinguish a third phase, punctuated by the European arrival, which may have intensified the signals of phase II, involving even more intensive agriculture, sedentism and demographic pressure, with a decrease in indigenous multilingualism due to the role of Spanish as *lingua franca* [76].

This scenario would mean that the integration hypothesis best describes the situation in phase I, while phase II, at least for some of the smaller language families and isolates, was increasingly consistent with the isolation hypothesis. This would then mean that both isolation and identity preservation during periods of contact have been involved in the maintenance of diversity in different phases: identity preservation in periods of equilibrium, isolation in periods of punctuation.

Zooming in on three individual groups, Puinave, Kamsá and Tikuna, we can additionally say that, even if there are general patterns to be discerned, this certainly does not mean that all groups behaved alike. In fact, the three isolates that we focused on in more detail show three different patterns.

Puinave shows signals that are compatible with the integration hypothesis. Situated in an area of intermediate endemism, Puinave is surrounded by a mix of larger (Arawakan) and smaller (Guahiban, Saliban) language families, shows signals of local convergence in their sociocultural profile, whereas it differs more from groups that live further away. More specifically, the cultural practices of the Puinave resemble those of Arawakan and East Tukanoan groups, particularly those of the Upper Rio Negro/Vaupés area. Linguistically speaking, there is evidence of, in particular, phonological convergence with surrounding languages, although there is also some signal that suggests connections to languages in the southeastern part of the NWA, which may be indicative of a historically more distant connection with groups spoken there, for which there is some tentative support [40].

Genetically, Puinave shows signals of contact throughout all shared IBD length categories, particularly with Arawakan- and Eastern-Tukanoan-speaking groups. The anthropological, linguistic and genetic results place Puinave within a network of groups that have been interacting for a long time, confirming the common practice of social/linguistic exogamy among different ethnolinguistic groups in a large area comprising the basins of the Vaupes, Rio Negro, and Orinoco Rivers [33,74]. Furthermore, ethnohistorical accounts show the existence of large networks of exchange, where Arawakans played a central role in the so-called Manoa macropolity, where multilingualism was commonplace, and connected diverse ethnicities over large geographical distances that were disrupted with the arrival of colonial powers [75,77].

Kamsá, situated in an area of intermediate to high endemism (i.e. closer to the prototype of a remnant island of high diversity), shows no clear signals of linguistic convergence with surrounding languages, including Inga. This is unexpected, since Inga is Kamsá's closest neighbour, both groups inhabiting the Sibundoy Valley and the eastern foothills of the Andes, where both the Putumayo and Caqueta Rivers originate. Genetically speaking, however, Kamsá consistently shares IBD blocks with Inga (figure 7), which is consistent with patterns of intermarriage between individuals of both groups and supported by a previous study based on the analysis of shared mtDNA sequences [78]. Interestingly, IBD sharing between Inga and Kamsá goes back to the oldest period of the IBD analysis 2500–1500 years ago. The arrival of Quechua in the area, and the subsequent language shifts to Quechuan that took place did not start until the fifteenth century [79]. This means that the earliest genetic interactions between Inga and Kamsá date back to a time where the group that is today associated with the Inga Quechua language, spoke a different, non-Quechuan language. This may partly help explain the discrepancy between the linguistic and genetic signals.

Tikuna, finally, spoken in an area of low endemism, shows little evidence of convergence in their sociocultural profile. Linguistically, Tikuna, like Puinave, shows a mixed signal. There is little evidence of morphosyntactic convergence, but the language does show signs of influence from its neighbours in its phonology. Communicative interaction with neighbouring groups is also evident in the lexicon, where Tupian and Quechuan loanwords have been identified [37]. Genetically speaking, Tikuna shows signals compatible with the isolation hypothesis during the most recent time period (0–500 ybp), since IBD sharing occurs exclusively within the group. During the time periods of 1500–500 and 2500–1500 ybp, we observe some sharing with neighbouring groups Yagua and Cocama, suggesting genetic contact with these groups. A further interesting observation from this analysis is the sharing of small (1–5 cM) IBD blocks with Murui and Uitoto. This is supported by f-statistics that show significant sharing of genetic drift between Tikuna, Murui and Uitoto (figure 7; electronic supplementary material, figure S4*a*). Although these groups live further north along the middle-Putumayo River, these results suggest that the ancestors of these three groups have interacted further back in the past (greater than 1500 ybp). For instance, the now-extinct sister language of Tikuna, Yuri, was recorded on the Caquetá River of Colombia not far from the area where the Murui, Uitoto and other Witoto-speaking groups are distributed [80], therefore making genetic contact between the ancestors of these groups a plausible situation.^16^

## Data Availability

Datasets and scripts are available at https://figshare.com/projects/isolates_focus/147958. Given the sensitive nature of the human genetic data generated in this study, genetic data will not be made publicly available, but deposited to the European Genome-Phenome Archive (EGA: https://ega-archive.org accession code EGAS00001006767). Access to the data will be granted by a Data Access Committee upon agreeing the conditions on the Data Access Agreement Form available upon request. The data are provided in the electronic supplementary material [81].

## Endnotes

1 All data on global distributions of languages and language families reported on in this paper are based on [7].

2 Although work on language classification is still ongoing, the wealth of data about Indigenous South American languages that has become available in the last few decades has given the language classifications a strong basis. We can therefore draw a solid conclusion that the genealogical diversity in South America is factually correct.

3 In this paper, we focus mainly on South America, and more specifically on the north-western part of the Amazon basin. This relates to a concentration of genealogical diversity there. Moreover, a considerable part of the North American Indigenous population stems from later migrations, making the American Paradox particularly pronounced for South America.

4 This is possibly consistent with a suggested period of about 30 000 years of isolation in Beringia before the first people moved into the Americas, suggested by several genetic studies [13–16].

5 This is possibly consistent with reports from other newly populated areas where diversification seems to have been rapid [17–19].

6 See [20, p. 84] for a similar statement.

7 Two major, and probably decisive differences between the Old and the New World, according to [22,23] were the lack of large domesticated animals and major cereals apart from maize in the New World.

8 It should be said that the geographical scope of Epps’ proposal is narrower than that of Nettle. Epps [11] focuses on the Upper Río Negro area, though she hypothesizes that her proposal may be extendable to the Amazon Basin more generally.

9 Depending on the dataset, different names may be used. In the text, we refer to the language as Kamsá, and to the people as Kamentsa, but some of the plots contain the name Camsá. This is also true for Tikuna, which may surface as Ticuna in some plots.

10 The genealogical relation between Tikuna and Yuri (and perhaps further groups) is still a matter of debate. We follow Glottolog [7] here in considering Tikuna and Yuri to form a genealogical unit (see [38,39] for supporting arguments).

11 Data preparation, analysis and visualization were done using the workflows implemented in the glottospace R package [41], which provides wrappers to functions of several other packages, including the cluster package for calculating distances [56], the vegan package [57] for performing NMDS and permanova, and ggplot2 [58] and plotly [59] for visualizing NMDS results.

12 The raw distances suggest that the Quechuan groups show some affinities with Awa Pit (Barbacoan), Kokama (Tupian), and especially Shiwiar and Huambisa (both Chicham).

13 See section S0 of the electronic supplementary material for the precise sample.

14 This is also corroborated by the heat map and the discussion based on the raw distances, provided in the electronic supplementary material, section S4 where the areas of greatest convergence are clearly family-internal.

15 In addition, making a distinction between phonological features and morphosyntactic features reveals that even languages that look similar may have different relations with their geographical neighbours. At this point, it is unclear what these subpatterns mean. The plots are given in the electronic supplementary material.

16 It is noteworthy that, although Tikuna is a language spoken by a large group of people living in the triple frontier between Colombia, Peru and Brazil along the Amazon River, with an intense movement of people and goods along its main course and its tributaries. Nevertheless, we do not observe high amounts of long IBD sharing with other groups, although this might reflect the fact that our sampling of Tikuna individuals was restricted to the town of Leticia in Colombia, and thus it would be interesting to investigate if there are local differences among Tikuna-speaking communities.
